# Nasal Carriage of Methicillin-Resistant *Staphylococcus Sciuri* Group by Residents of an Urban Informal Settlement in Kenya

**DOI:** 10.24248/eahrj.v7i1.711

**Published:** 2023-07-12

**Authors:** Charchil Ayodo, Robert Mugoh, Teresa Ita, Collins Ouma, Moureen Jepleting, Beatrice Oduor, Bernard Guyah, Sylvia Omulo

**Affiliations:** a Department of Biomedical Sciences and Technology, Maseno University, Kenya; b Washington State University Global Health-Kenya, Nairobi, Kenya; c Paul G. Allen School for Global Health, Washington State University, Pullman, WA, US; d University of Nairobi Institute of Tropical and Infectious Diseases, Nairobi, Kenya.

## Abstract

**Background:**

The *Staphylococcus sciuri* group constitutes animal-associated bacteria but can comprise up to 4% of coagulase-negative staphylococci isolated from human clinical samples. They are reservoirs of resistance genes that are transferable to *Staphylococcus aureus* but their distribution in communities in sub-Saharan Africa is unknown despite the clinical importance of methicillin-resistant *S. aureus*.

**Objectives:**

We characterised methicillin-resistant *S. sciuri* group isolates from nasal swabs of presumably healthy people living in an informal settlement in Nairobi to identify their resistance patterns, and carriage of two methicillin resistance genes.

**Method:**

Presumptive methicillin-resistant *S. sciuri* group were isolated from HardyCHROM™ methicillin-resistant *S. aureus* media. Isolate identification and antibiotic susceptibility testing were done using the VITEK^®^2 Compact. DNA was extracted using the ISOLATE II genomic kit and polymerase chain reaction used to detect *mecA* and *mecC* genes. Results: Of 37 presumptive isolates, 43% (16/37) were methicillin-resistant including - *S. sciuri* (50%; 8/16), *S. lentus* (31%; 5/16) and *S. vitulinus* (19%; 3/16). All isolates were susceptible to ciprofloxacin, gentamycin, levofloxacin, moxifloxacin, nitrofurantoin and tigecycline. Resistance was observed to clindamycin (63%), tetracycline (56%), erythromycin (56%), sulfamethoxazole/trimethoprim (25%), daptomycin (19%), rifampicin (13%), doxycycline, linezolid, and vancomycin (each 6%). Most isolates (88%; 14/16) were resistant to at least 2 antibiotic combinations, including methicillin. The *mecA* and *mecC* genes were identified in 75% and 50% of isolates, respectively.

**Conclusion:**

Colonizing *S. sciuri* group bacteria can carry resistance to methicillin and other therapeutic antibiotics. This highlights their potential to facilitate antimicrobial resistance transmission in community and hospital settings. Surveillance for emerging multidrug resistant strains should be considered in high transmission settings where human-animal interactions are prevalent. Our study scope precluded identifying other molecular determinants for all the observed resistance phenotypes. Larger studies that address the prevalence and risk factors for colonization with *S. sciuri* group and adopt a one health approach can complement the surveillance efforts.

## INTRODUCTION

The *Staphylococcus sciuri* group (*S. sciuri, S. lentus*, and *S. vitulinus*) consists of coagulase-negative staphylococci that are distinguishable from other staphylococci by a positive oxidase test.^1^ These bacteria can be isolated from the environment, animals, and dairy products. People may be colonized in the nasopharynx and skin^2^ following repeated contact with colonized livestock and pets^2^ or through contact with food animal products.^3^ Nevertheless, human infection with the *S. sciuri* group does occur; they can constitute up to 4% of coagulase-negative staphylococci isolated from clinical samples, and can cause endocarditis, peritonitis, septic shock, urinary tract infections, pelvic inflammatory disease, and wound infections.^1^ Over the last decade, oxacillin/methicillin-resistant staphylococcal strains have emerged, increasing the medical relevance of the *S. sciuri* group.^3^ These bacteria can receive^5^ and transfer resistance genes to human and animal pathogens such as *Staphylococcus aureus*^4^ and can carry a *mecA* gene that is closely related to the methicillin-resistant *S. aureus* (MRSA) *mecA* gene.^4^ The *mecA* gene encodes broad-spectrum beta-lactam resistance.^4^ A novel *mecA* homolog – *mecC* – which also confers resistance to methicillin, has emerged in staphylococci isolated from animals, humans, and the environment.^6^

We characterised *S. sciuri* group isolates from nasal swabs collected from presumably healthy people to determine their antibiotic resistance profiles and the proportion harbouring *mecA* and *mecC* genes.

## MATERIALS AND METHODS

During a 2019 population–based study on antimicrobial resistance in communities and hospitals in Kenya (KNH/UoN ERC# P164/03/2018), which targeted colonizing MRSA strains from presumably healthy people in Kibera—an informal settlement in Nairobi— we unexpectedly cultured *S. sciuri* group bacteria on HardyCHROM™ MRSA media (Hardy diagnostics, CA) -chromogenic media that can isolate and differentiate *S. aureus* and other staphylococci.^7^ Nasal swabs were plated on HardyCHROM™ MRSA media, followed by incubation at 37°C overnight (18–24 hrs). After incubation, single small blue colonies, presumptively identified as methicillin-resistant *S. sciuri* group, were collected from each positive agar plate and sub-cultured on tryptic soy agar plates (KEMRI Production Department, Nairobi), then incubated overnight.

Species identification and antibiotic susceptibility testing (AST) of the purified (sub-cultured) isolates were done using the VITEK®2 Compact (Biomerieux, Marcy-l'Étoile). Isolates that were either oxacillin resistant and/or cefoxitin-screen positive were regarded as methicillin-resistant. Bacterial suspensions were prepared by adding discreet colonies into 3 mL of 0.5% (w/v) normal saline and adjusting turbidity to 0.5 McFarland. Isolate suspensions were tested against 15 antibiotics i.e., ciprofloxacin (≥4 mg/L), clindamycin (≥4 mg/L), daptomycin (8 mg/L), doxycycline (≥16 mg/L), erythromycin (≥8 mg/L), gentamycin (≥16 mg/L), levofloxacin (≥8 mg/L), linezolid (≥8 mg/L), moxifloxacin (≥2 mg/L), nitrofurantoin (≥128 mg/L), rifampicin (≥4 mg/L), sulfamethoxazole/trimethoprim (≥4/76 mg/L), tetracycline (≥16 mg/L), tigecycline (2 mg/L) and vancomycin (≥32 mg/L). Minimum inhibitory concentration values were interpreted following the 2020 Clinical Laboratory Standards Institute standards.^8^ Isolates with intermediate resistance were considered susceptible. We defined multidrug resistance as resistance to at least one antibiotic in three or more antibiotic classes.

DNA extraction from confirmed isolates was done using the ISOLATE II genomic kit (Bioline, FL) following manufacturer instructions, and stored at -20 °C until tested. The presence of *mecA* and *mecC* genes was determined using the *S. aureus mecA* and *mecC* primers on the VeritiPro Thermal Cycler (Thermo Fisher scientific, MA). Separate reaction mixes were prepared for mecA and *mecC*. Each 25 μL reaction mix consisted of 0.5 μL of each [0.2 μM] primer pair i.e., *mecA*F—5′GT AGA AAT GAC TGA ACG TCC GAT AA3′, *mecA*R—5′CCA ATT CCA CAT TGT TTC GGT CTA A3′ (310 bp), and *mecC*F—5′G CTC CTA ATG CTA ATG CA3′, *mecC*R—5′TAA GCA ATA ATG ACT ACC3′ (304 bp), respectively, 12.5 μL of 2X MyTaq™ Red Mix (Bioline, FL), 2 μL of DNA template and 9.5 μL of PCR-grade water. Thermocycling proceeded as follows: 95 °C, 1 min; 95 °C, 15 s; 51 °C, 15 s (30 cycles), 72 °C, 10 s. Amplified DNA (5 μL) was stained with 2 μL cyber-green dye and run in a 1% agarose gel alongside a 1 kB ladder. The gel was run in 5X Tris-acetate EDTA buffer (90 volts, 65mA and 6 watts) for 35 min. Two positive controls, ATCC 33591 (MRSA) and ATCC BAA 2312 (*S. aureus) -* were included to confirm *mecA* and *mecC* gene fragments. ATCC 25922 (*E. coli*) was used as the negative control. Bands corresponding to 310 bp and 304 bp under UV light confirmed the presence of *mecA* and *mecC* genes, respectively. The Qubit^TM^ 4 Fluorometer was used to measure DNA concentration to ensure concentrations above 2.5 ng/μL. DNA quality was confirmed by the absence of extraneous bands during gel electrophoresis.

### Ethical Approval

The isolates analysed in this study were identified during sample processing for the ARCH study. The ARCH study was approved by the KNH/UoN ERC (# P164/03/2018).

## RESULTS

In total, 37 presumptive methicillin–resistant *S. sciuri* group isolates were collected from HardyCHROM™ MRSA plates. Of these, 43% (16/37) were positively identified by the Vitek2 as methicillin-resistant *S. sciuri* group i.e., *S. sciuri* (50%; 8/16), *S. lentus* (31%; 5/16) and *S. vitulinus* (19%; 3/16). All isolates were susceptible to ciprofloxacin, gentamycin, levofloxacin, moxifloxacin, nitrofurantoin and tigecycline. Conversely, more than half of all isolates were resistant to clindamycin (63%), erythromycin (56%), and tetracycline (56%), with less than one-third resistant to the remaining antibiotics (Table 1).

**TABLE 1:** Antibiotic Resistance among Methicillin-Resistant S. Sciuri Group Isolates

Antibiotic tested	S. sciuri (n = 8)	S. lentus (n = 5)	S. vitulinus (n = 3)	S. sciuri gp (n = 16)
Clindamycin	6 (75%)	4 (80%)	0 (0%)	10 (63%)
Daptomycin	1 (13%)	2 (40%)	0 (0%)	3 (19%)
Doxycycline	1 (13%)	0 (0%)	0 (0%)	1 (6%)
Erythromycin	7 (88%)	0 (0%)	2 (67%)	9 (56%)
Linezolid	1 (13%)	0 (0%)	0 (0%)	1 (6%)
Rifampicin	2 (25%)	0 (0%)	0 (0%)	2 (13%)
Sulfamethoxazole-trimethoprim	3 (38%)	1 (20%)	0 (0%)	4 (25%)
Tetracycline	6 (75%)	0 (0%)	3 (100%)	9 (56%)
Vancomycin	1 (13%)	0 (0%)	0 (0%)	1 (6%)

Most isolates (88%; 14/16) were resistant to several antibiotic combinations in addition to methicillin. Four (25%) were resistant to one antibiotic, four (25%) to two antibiotics, four (25%) to three antibiotics, one (6%) to five antibiotics and one (6%) to eight antibiotics. Clindamycin-daptomycin (CLI-DAP), clindamycin-erythromycin-tetracycline (CLI-ERY-TET) and erythromycin-tetracycline (ERY-TET) were common multidrug resistant phenotypes (Table 2).

**TABLE 2:** Distribution of Study Participants According to Age and Sex

Isolate	Species	mecA	mecC	CLI	DAP	DOX	ERY	LZD	RIF	SXT	TET	VAN	Resistance phenotype
1	S. sciuri	Y	Y	+	-	-	+	-	-	+	-	-	CLI-ERY-SXT
2	S. sciuri	N	Y	-	-	-	+	-	-	+	+	-	ERY-SXT-TET
3	S. sciuri	Y	N	+	+	-	+	+	+	-	+	+	CLI-DAP-DOX-ERY-LNZ-RIF-TET-VAN
4	S. sciuri	Y	Y	+	-	-	-	-	-	-	-	-	CLI
5	S. sciuri	Y	Y	+	-	-	+	-	-	-	+	-	CLI-ERY-TET
6	S. sciuri	Y	N	-	-	-	+	-	-	-	+	-	ERY-TET
7	S. sciuri	Y	Y	+	-	-	+	-	-	-	+	-	CLI-ERY-TET
8	S. lentus	Y	N	-	-	-	-	-	-	-	-	-	-
9	S. lentus	Y	N	+	+	-	-	-	-	-	-	-	CLI-DAP
10	S. lentus	N	N	+	+	-	-	-	-	-	-	-	CLI-DAP
11	S. lentus	N	Y	+	-	-	-	-	-	-	-	-	CLI
12	S. vitulinus	Y	N	-	-	-	-	-	-	-	+	-	TET
13	S. vitulinus	Y	N	-	-	-	+	-	-	-	+	-	ERY-TET
14	S. lentus	N	Y	-	-	-	-	-	-	-	-	-	-
15	S. vitulinus	Y	N	-	-	-	-	-	-	-	+	-	TET
16	S. sciuri	Y	Y	+	-	-	+	-	+	+	+	-	CLI-ERY-RIF-SXT-TET
	# R	-	-	10	3		9	1	2	4	10	1	
	% R	-	-	56	17	1	50	6	11	22	56	6	

CLI, clindamycin; DAP, daptomycin; DOX, doxycycline; ERY, erythromycin; LZD, linezolid; RIF, rifampicin; SXT, sulfamethoxazole-trimethoprim; TET, tetracycline; VAN, vancomycin. Resistant (+); Susceptible (-); Y, present; N, absent; R, Resistant.

The *mec*A gene was identified in 75% (12/16) of isolates, while *mecC* in 50% (8/16). Overall, 44% (7/16) of isolates carried the *mecA* gene only, 19% (3/16) carried *mecC* only, 31% (5/16) carried *mecA* and *mecC*, while 6% (1/16) had neither gene (Table 2).

## DISCUSSION

Focus on the *S. sciuri* group bacteria has increased in recent years owing to their implication in opportunistic human and veterinary infections^9,10^ food contamination,^11,12^ and potential for zoonotic transmission.^13^ These bacteria are natural reservoirs of methicillin resistance genes, which can be transferred to *S. aureus*—an important human and animal pathogen—and can carry virulence genes that promote pathogenicity in coagulase-negative staphylococci.^10,14^

Colonising strains of methicillin-resistant *S. sciuri* group in the nasal cavities of presumptively healthy individuals in sub-Saharan Africa have not widely been reported. Consequently, little is known about the distribution of these bacteria within communities, despite their potential to transfer resistance genes to pathogenic staphylococci. Previous studies have reported low prevalence (~5%) of *S. sciuri* infections in hospitals—presumably cross-transmitted between patients and healthcare workers^15^— and in communities, presumably transmitted via close contact with animals.^2^ It is likely that colonizing strains, as those found in our study, are transmitted via bioaerosols, with dust mediating the transfer of environmental bacteria when inhaled.^16^ Informal settlements are commonly characterized by poor environmental hygiene, which can facilitate the thriving of *S. sciuri* group bacteria. Data from the parent study indicate that 81% of sampled households keep domestic animals, the majority (76%) of which are dogs^17^—a known reservoir of *S. sciuri.*

The susceptibility of *S. sciuri* group isolates to ciprofloxacin, gentamycin, levofloxacin, moxifloxacin, nitrofurantoin and tigecycline is consistent with another study.^15^ This suggests that these antibiotics can be used in the management of infections caused by *S. sciuri* group bacteria. Conversely, we identified an isolate that was resistant to eight antibiotics, including vancomycin—used to treat *S. sciuri* infections^16^—highlighting the potential public health threat that can arise if such strains become amplified in communities and hospitals. While the clinical significance of *S. sciuri* group may be unappreciated, the capacity of these bacteria to carry multidrug resistance is well established^2,18^ and has been reported in clinical studies in Serbia^3,15^ and Nigeria.^19^

The observed distribution of *mecA* and *mecC* genes in our sample is consistent with findings from a study in Tunisia.^13^ Resistance to clindamycin can be mediated via the *erm* gene, which is located on transposon Tn554, which has insertion sites in the *Staphylococcus* spp. chromosome, where *mecA* and *mecC* genes are contained,^20^ and may explain the observed resistance to clindamycin among the isolates with both *mecA* and *mecC* genes. Tetracycline resistance was common among isolates with the *mecA* gene, as demonstrated by other studies,^21^ suggesting that *tet* and *mecA* genes may be located on the same genetic element. The absence of *mecA* and *mecC* genes in one isolate despite its resistance to clindamycin, daptomycin and methicillin suggests that other resistance elements mediate resistance to beta-lactams and other antibiotics within the group of bacteria.^13,22^

One limitation of this study was its limited scope which precluded identifying other molecular determinants for the observed resistance phenotypes. Larger studies that address the prevalence and risk factors for colonization with *S. sciuri* group and adopt a one health approach can complement surveillance efforts.

## CONCLUSION

Nasal colonization with methicillin-resistant *S. sciuri* group bacteria appears low in the population studied and may not be mediated by companion animals (e.g., dogs) which were prevalent in this population. Nevertheless, these bacteria are resistant to medically important antibiotics and carry important resistance genes, presenting a potential AMR threat.
